# Hyperactivated endolysosomal trafficking in melanoma

**DOI:** 10.18632/oncotarget.3141

**Published:** 2015-02-04

**Authors:** Direna Alonso-Curbelo, Maria S. Soengas

**Affiliations:** Melanoma Laboratory, Molecular Oncology Programme, Centro Nacional de Investigaciones Oncológicas (CNIO), Madrid, Spain

Cutaneous melanoma is an increasingly frequent solid tumor that accounts for 80% of skin cancer-related deaths. Characterized by a complex and heterogeneous genetic background and a notorious functional plasticity, melanoma has shifted from a “black box” to a paradigm of how basic research can be successfully translated into significant improvements of patient prognosis [1]. Indeed, the identification of mutations in key oncogenic pathways (most frequently hyperactivating the BRAF>MEK signaling cascade) has paved the way to the development of genetically-targeted therapies [1]. Similarly, the discovery of intrinsic brakes to immune activation (i.e. involving the CTLA-4, PD1 or PD-L1 immune checkpoints) has also represented a clinical breakthrough in this disease [2]. Response rates, however are still incomplete [2]. Therefore, the field remains in need of a better understanding of the mechanistic basis underlying tumor progression and resistance to therapy.

The search for tumor drivers and druggable vulnerabilities in melanoma has traditionally involved “within lineage” genomic studies. Specifically, analyses of benign and/or malignant melanocytic lesions, or comparisons of normal and tumoral melanocytic cells have yielded seminal discoveries such as the pro-oncogenic alteration of *BRAF* mentioned above, as well as genetic changes in *NRAS, KIT, TERT, RAC or ERBB4*, among others [3]. However, these are just a small fraction of the still to be understood over 80,000 mutations that have been reported in melanoma specimens to date [3]. An alternative and less explored strategy for the discovery of tumorigenic factors is to perform “across lineage” analyses (i.e. assessing different cancer types). Such approach led to the discovery of microphthalmia-associated transcription factor (MITF) as a key lineage-specific oncogene in melanoma. Nevertheless, although *MITF* is amplified in a subset of melanomas, it is also frequently repressed post-transcriptionally [4]. This raises the possibility of other factors fostering melanoma progression in a lineage-specific but MITF-independent manner.

To identify new oncogenes selectively activated in melanoma, we performed gene set enrichment analyses (GSEA) on multitumor transcriptomic datasets. Scoring for processes, pathways or cellular compartments over-represented in melanoma, we identified a large cluster of lysosome- and endolysosome-associated genes that distinguished this disease from over 35 different cancer types [5]. These results were curious as the endolysosomal pathway is ubiquitously active in normal and malignant cells. In particular, one of the melanoma-overexpressed genes that caught our attention was the RAB7A small GTPase. RAB7A (herein referred to as RAB7 for simplicity) was attractive for its key roles in lysosomal-dependent degradation of autophagosomes and endosomes [6], none of which were expected to be controlled in a tumor type-specific manner.

**Figure 1:**
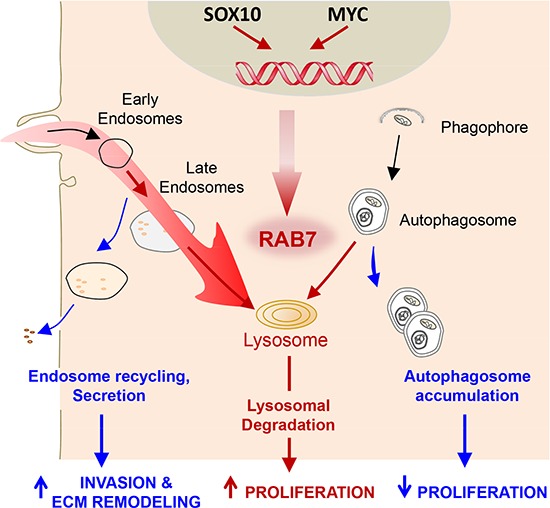
Regulation and function of RAB7 in melanoma In this tumor type, RAB7 levels are controlled by the melanocytic lineage-specification factor SOX10 and the MYC oncogene. High RAB7 levels (red lines) allow for an efficient lysosomal-dependent degradation of endocytic and autophagic vesicles, ensuring an active proliferation of melanoma cells. Tuning down RAB7 levels in melanoma cells (blue lines) can shift the fate of endosomal cargo (including various pro-invasive factors, among other proteins) from degradation to recycling or secretion, favoring metastatic dissemination.

Histological and functional analyses in cell lines, supported by further validation in genetically modified mice, demonstrated that RAB7 was highly induced already from early stages of melanoma development, being selectively required to sustain tumor cell proliferation. This dependency was linked to a strict requirement of RAB7 to counteract, by lysosomal degradation, a hyperactive influx of large endosomal vesicles (macropinosomes) generated at the plasma membrane of melanoma cells (see summary in Figure 1). Interestingly, the melanoma-selective accumulation and requirement of RAB7 were found independent from the lineage-specific oncogene MITF. Instead, we discovered RAB7 to be transactivated by SOX10 (an early driver of the melanocytic lineage) and by the oncogene MYC [5], illustrating new mechanisms controlling endosomal trafficking.

Intriguingly, while RAB7 levels were higher in all melanoma specimens characterized than in their surrounding stroma, the levels of this protein were not constant during tumor progression. Thus, invasive primary melanomas showed a progressively diminished RAB7 expression towards their dermal front, a feature that correlated with an increased risk for metastasis [5]. The dual roles of RAB7, namely (i) enhanced cell division when highly expressed, and (ii) metastasis when tuned down, reflected a switch in the “fate” of the cargo of macropinosomes and other vesicular structures, from lysosomal degradation towards recycling or secretion, respectively (Figure 1). Importantly, this derailed vesicle trafficking translated into marked changes in the transcriptome, proteome and secretome of melanoma cells. These pleiotropic effects, triggered without direct binding to DNA, further differentiate RAB7 from classical lineage-specific tumor drivers, which most frequently correspond to transcription factors.

Time-lapse analyses of vesicular trafficking demonstrated that, different from melanoma cells, normal melanocytes had a rather silent RAB7-regulated macropinocytosis. These results are exciting as they offer the opportunity of therapeutic intervention at different levels: (i) exploiting differential rates of macropinocytosis in normal vs tumor cells for cancer-targeted drug delivery, and (ii) promoting tumor cell death by “exhaustion”, exacerbating endogenous endolysosomal degradation, resulting in depletion of critical cellular components. We have demonstrated that these two features are in fact possible with bioavailable complexes of long dsRNA and polycationic carriers (termed as BO-110 for simplicity). BO-110 forms nanoparticle size clusters (100–150 nm), which we had previously found to kill melanoma cells effectively with minimal secondary toxicities *in vivo* [7]. The negligible macropinocytic activity of normal melanocytes provides now a plausible molecular explanation as to why these (and other normal cells) can be spared from the incorporation of BO-110. Moreover, electron microscopy revealed that BO-110 promoted a massive hyperactivation of RAB7 and its regulated endolysosomal traffic followed by melanoma cell collapse by hyperactivated autophagy and apoptosis [7].

These newly identified roles and regulatory mechanisms of RAB7, not only provide insight into lineage-specific mechanisms underlying melanoma progression, but also reveal intrinsic vulnerabilities of the endolysosomal machinery that can be harnessed for tumor-selective drug delivery and cell death. We anticipate that further untangling of vesicle trafficking routes and their particular context-dependencies may prove relevant for the management of cancer and other pathologies such as neurodegenerative diseases, in which RAB7 and other trafficking orchestrators are frequently deregulated.
